# Amicarbazone

**DOI:** 10.1107/S1600536813007782

**Published:** 2013-03-28

**Authors:** Manpreet Kaur, Ray J. Butcher, Jerry P. Jasinski, H. S. Yathirajan, B. P. Siddaraju

**Affiliations:** a Department of Studies in Chemistry, University of Mysore, Manasagangotri, Mysore 570 006, India; b Department of Chemistry, Howard University, 525 College Street NW, Washington, DC 20059, USA; c Department of Chemistry, Keene State College, 229 Main Street, Keene, NH 03435-2001, USA; d Department of Chemistry, G. Madegowda Institute of Technology, Bharathinagara 571 422, India

## Abstract

Three independent mol­ecules comprise the asymmetric unit of the title compound, C_10_H_19_N_5_O_2_, (systematic name: 4-amino-*N*-*tert*-butyl-3-isopropyl-5-oxo-4,5-dihydro-1*H*-1,2,4-triazole-1-carboxamide) . In all three mol­ecules, the triazole ring and the carboxamide group are almost coplanar [within 4.0–5.9 (9)°], particularly because of the formation of an intra­molecular N—H⋯O hydrogen bond. On other hand, the orientation of the isopropyl group varies significantly from mol­ecule to mol­ecule. The crystal packing is dominated by N—H⋯O and N—H⋯N hydrogen bonds, which connect the mol­ecules into infinite chains along [010].

## Related literature

For herbicidal properties of amicarbazone and for its preparation, see: Dayan *et al.* (2009 ▶); Diehr (1998 ▶). For related structures, see: Crockett *et al.* (2004 ▶); Dupont *et al.* (1991 ▶); Heng *et al.* (2006 ▶). For standard bond lengths, see: Allen *et al.* (1987 ▶).
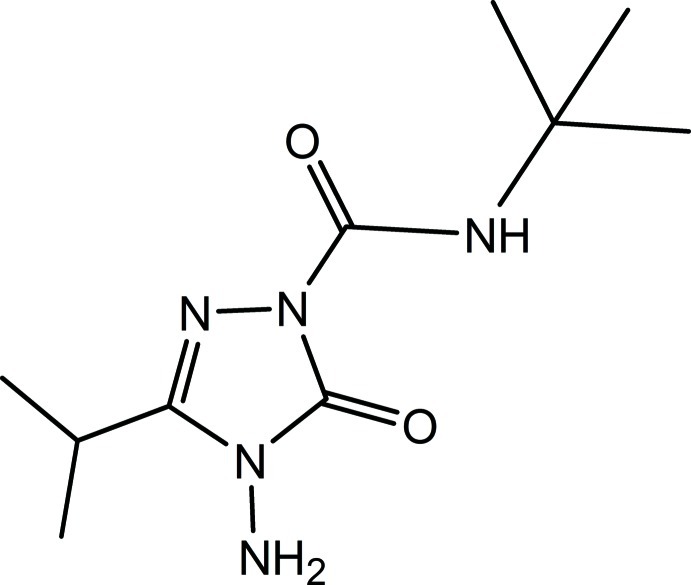


## Experimental

### 

#### Crystal data

C_10_H_19_N_5_O_2_
*M* *_r_* = 241.30
Triclinic, 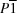

*a* = 11.0298 (2) Å
*b* = 12.2135 (4) Å
*c* = 14.8542 (4) Å
α = 92.244 (2)°
β = 95.7020 (19)°
γ = 93.379 (2)°
*V* = 1985.65 (9) Å^3^
*Z* = 6
Cu *K*α radiation
μ = 0.72 mm^−1^
*T* = 123 K
0.35 × 0.25 × 0.15 mm

#### Data collection

Agilent Xcalibur (Ruby, Gemini) diffractometer
Absorption correction: multi-scan (*CrysAlis PRO* and *CrysAlis RED*; Agilent, 2012 ▶) *T*_min_ = 0.791, *T*_max_ = 1.000
13380 measured reflections
7959 independent reflections
7592 reflections with *I* > 2σ(*I*)
*R*_int_ = 0.019

#### Refinement

*R*[*F* ^2^ > 2σ(*F* ^2^)] = 0.044
*wR*(*F* ^2^) = 0.115
*S* = 1.05
7959 reflections
499 parameters
H atoms treated by a mixture of independent and constrained refinement
Δρ_max_ = 0.33 e Å^−3^
Δρ_min_ = −0.28 e Å^−3^

### 

Data collection: *CrysAlis PRO* (Agilent, 2012 ▶); cell refinement: *CrysAlis PRO*; data reduction: *CrysAlis PRO*; program(s) used to solve structure: *SHELXS97* (Sheldrick, 2008 ▶); program(s) used to refine structure: *SHELXL97* (Sheldrick, 2008 ▶); molecular graphics: *SHELXTL* (Sheldrick, 2008 ▶); software used to prepare material for publication: *SHELXTL*.

## Supplementary Material

Crystal structure: contains datablock(s) global, I. DOI: 10.1107/S1600536813007782/ld2097sup1.cif

Structure factors: contains datablock(s) I. DOI: 10.1107/S1600536813007782/ld2097Isup2.hkl

Supplementary material file. DOI: 10.1107/S1600536813007782/ld2097Isup3.cml

Additional supplementary materials:  crystallographic information; 3D view; checkCIF report

## Figures and Tables

**Table 1:** Hydrogen-bond geometry (Å, °)

*D*—H⋯*A*	*D*—H	H⋯*A*	*D*⋯*A*	*D*—H⋯*A*
N2*A*—H3*N*1⋯O1*C*	0.904 (19)	2.220 (19)	3.0832 (14)	159.6 (15)
N2*A*—H3*N*2⋯N3*B*	0.91 (2)	2.40 (2)	3.2251 (15)	150.9 (15)
N2*A*—H3*N*2⋯O2*B*	0.91 (2)	2.483 (19)	3.2492 (14)	141.7 (15)
N5*A*—H4*AB*⋯O1*A*	0.88	2.05	2.7770 (14)	140
N2*B*—H3*B*2⋯N3*C* ^i^	0.929 (19)	2.233 (19)	3.1619 (15)	179.1 (15)
N2*B*—H3*B*1⋯O1*B* ^ii^	0.89 (2)	2.27 (2)	3.1356 (15)	163.1 (16)
N5*B*—H4*BB*⋯O1*B*	0.88	2.04	2.7655 (14)	139
N2*C*—H3*C*2⋯O1*A*	0.906 (18)	2.171 (18)	2.9455 (15)	143.1 (15)
N2*C*—H3*C*1⋯O2*B*	0.88 (2)	2.41 (2)	3.0718 (14)	132.8 (16)
N5*C*—H4*CB*⋯O1*C*	0.88	2.05	2.7721 (13)	139

Symmetry codes: (i) 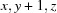; (ii) 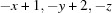.

**Fig. 1.**
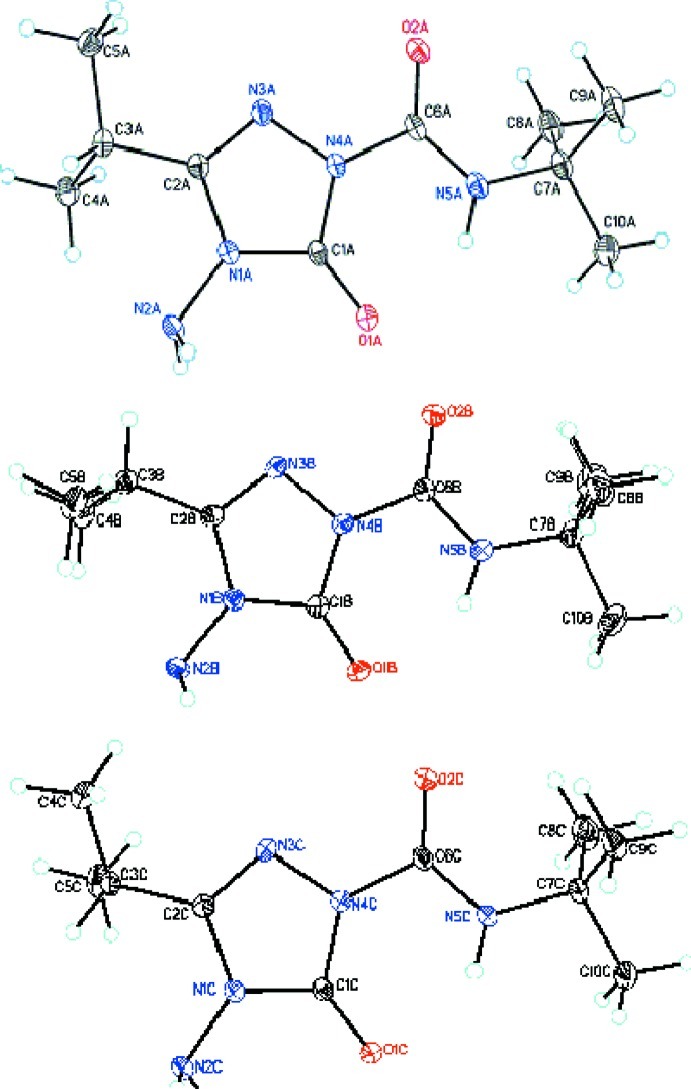
Molecular structure of the three independent molecules in the title compound showing the atom labeling scheme and 30% probability displacement ellipsoids.

**Fig. 2.**
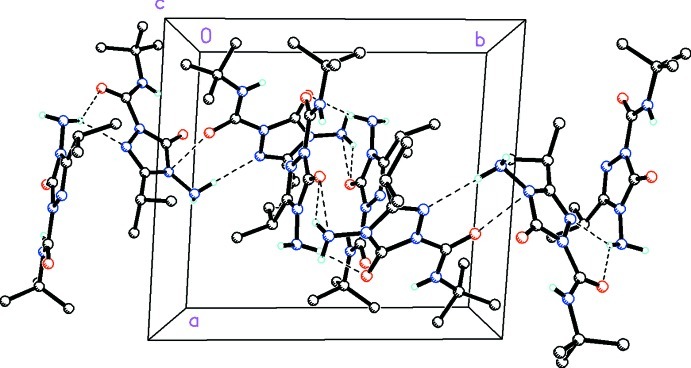
Packing diagram of the title compound viewed along the c axis. Dashed lines indicate N—H···O intramolecular hydrogen bonds and weak N—H···O, N—H···N intermolecular interactions which form a network of infininte 1-D chains along [010]. H atoms not involved in hydrogen bonds or weak intermolecular interactions have been removed for clarity.

## supplementary crystallographic information

### Comment

Amicarbazone (IUPAC name: 4-Amino-N-(tert-butyl)-4,5-dihydro-3- (iso-propyl)-5-oxo-1H-1,2,4-triazole-1-carboxamide) is a new triazolinone herbicide with a broad spectrum of weed control. The phenotypic responses of sensitive plants exposed to amicarbazone include chlorosis, stunted growth, tissue necrosis, and death. Its efficacy as both a foliar- and root-applied herbicide suggests that absorption and translocation of this compound is very rapid. This new herbicide is a potent inhibitor of photosynthetic electron transport, inducing chlorophyll fluorescence and interrupting oxygen evolution ostensibly via binding to the Qb domain of photosystem II (PSII) in a manner similar to the triazines and the triazinones classes of herbicides (Dayan *et al.*, 2009). In view of the importance of amicarbazone derivatives, this paper reports the crystal and molecular structure of the title compound, (I), C_10_H_19_N_5_O_2_.

In (I), three independent molecules (A,B,C) crystallize in the asymmetric unit (Fig. 1). The dihedral angle between the mean planes of the triazole ring and carboxamide group (N4/C6/O2/N5) for each of the molecules lie between 4.0 (6)° and 5.9 (9)°. In each of the three molecules the torsion angles of the tert-butyl groups are quite similar [C6/N5/C7/C8 = -56.76 (17)° (A), 64.18 (17)° (B), -57.90 (17)° (C); C6/N5/C7/C9 = 66.03 (16)° (A), -57.99 (19)° (B), 64.87 (16)° (C); C6/N5/C7/C10 = -175.58 (12)° (A), -177.48 (19)° (B), -177.61 (13)° (C)] while those of the isopropyl group vary significantly [N3/C2/C3/C4 = 111.38(14° (A), 120.86 (14)° (B), 8.92 (17)° (C); N3/C2/C3/C5 = -11.65 (17)° (A), -113.15 (14)° (B), 88.83 (15)° (C)]. In the crystal molecular packing is dominated by N—H···O and N—H···N intermolecular interactions (Table 1) forming a network of infinite 1-D chains along [010] (Fig. 2).

### Experimental

Amicarbazone was prepared according to the patent procedure (Diehr, 1998). The resulting compound was recrystallized from methyl t-butyl ether by slow evaporation (M.P.: 373–378 K).

### Refinement

The amine H-atoms (H3N1, H3N2, H3B1, H3B2, H3C1, H3C2) were located by a difference map and refined isotropically. All of the remaining H atoms including the amide H atoms, were placed in their calculated positions and then refined using the riding model with Atom—H lengths of 1.00Å (CH), 0.98Å (CH_3_) or 0.88Å (NH). Isotropic displacement parameters for these atoms were set to 1.18-1.20 (CH, NH) or 1.48-1.50 (CH_3_) times *U*_eq_ of the parent atom. All of the methyl groups were refined in idealized positions with their rotation angle optimized (AFIX 137).

### Crystal data

**Table:**

C_10_H_19_N_5_O_2_	*Z* = 6
*M**_r_* = 241.30	*F*(000) = 780
Triclinic, *P*1	*D*_x_ = 1.211 Mg m^−^^3^
Hall symbol: -P 1	Cu *K*α radiation, λ = 1.54184 Å
*a* = 11.0298 (2) Å	Cell parameters from 11341 reflections
*b* = 12.2135 (4) Å	θ = 3.0–75.4°
*c* = 14.8542 (4) Å	µ = 0.72 mm^−^^1^
α = 92.244 (2)°	*T* = 123 K
β = 95.7020 (19)°	Prism, colorless
γ = 93.379 (2)°	0.35 × 0.25 × 0.15 mm
*V* = 1985.65 (9) Å^3^	

### Data collection

**Table:**

Agilent Xcalibur (Ruby, Gemini) diffractometer	7959 independent reflections
Radiation source: Enhance (Cu) X-ray Source	7592 reflections with *I* > 2σ(*I*)
Graphite monochromator	*R*_int_ = 0.019
Detector resolution: 10.5081 pixels mm^-1^	θ_max_ = 75.6°, θ_min_ = 3.0°
ω scans	*h* = −13→9
Absorption correction: multi-scan (*CrysAlis PRO* and *CrysAlis RED*; Agilent, 2012)	*k* = −13→15
*T*_min_ = 0.791, *T*_max_ = 1.000	*l* = −17→18
13380 measured reflections	

### Refinement

**Table:**

Refinement on *F*^2^	Primary atom site location: structure-invariant direct methods
Least-squares matrix: full	Secondary atom site location: difference Fourier map
*R*[*F*^2^ > 2σ(*F*^2^)] = 0.044	Hydrogen site location: inferred from neighbouring sites
*wR*(*F*^2^) = 0.115	H atoms treated by a mixture of independent and constrained refinement
*S* = 1.05	*w* = 1/[σ^2^(*F*_o_^2^) + (0.0642*P*)^2^ + 0.6558*P*] where *P* = (*F*_o_^2^ + 2*F*_c_^2^)/3
7959 reflections	(Δ/σ)_max_ < 0.001
499 parameters	Δρ_max_ = 0.33 e Å^−^^3^
0 restraints	Δρ_min_ = −0.28 e Å^−^^3^

### Special details

**Table:**

Geometry. All esds (except the esd in the dihedral angle between two l.s. planes) are estimated using the full covariance matrix. The cell esds are taken into account individually in the estimation of esds in distances, angles and torsion angles; correlations between esds in cell parameters are only used when they are defined by crystal symmetry. An approximate (isotropic) treatment of cell esds is used for estimating esds involving l.s. planes.
Refinement. Refinement of *F*^2^ against ALL reflections. The weighted *R*-factor *wR* and goodness of fit *S* are based on *F*^2^, conventional *R*-factors *R* are based on *F*, with *F* set to zero for negative *F*^2^. The threshold expression of *F*^2^ > σ(*F*^2^) is used only for calculating *R*-factors(gt) *etc*. and is not relevant to the choice of reflections for refinement. *R*-factors based on *F*^2^ are statistically about twice as large as those based on *F*, and *R*- factors based on ALL data will be even larger.

### Fractional atomic coordinates and isotropic or equivalent isotropic displacement parameters (Å^2^)

**Table:**

	*x*	*y*	*z*	*U*_iso_*/*U*_eq_	
O1A	0.51333 (8)	0.54857 (7)	0.27512 (6)	0.02404 (19)	
O2A	0.79068 (9)	0.61028 (9)	0.49492 (7)	0.0338 (2)	
N1A	0.40274 (9)	0.61234 (8)	0.39102 (7)	0.0195 (2)	
N2A	0.28358 (9)	0.60744 (9)	0.34749 (8)	0.0228 (2)	
H3N1	0.2623 (16)	0.5362 (16)	0.3316 (12)	0.032 (4)*	
H3N2	0.2814 (17)	0.6526 (16)	0.2999 (13)	0.035 (4)*	
N3A	0.55089 (9)	0.64321 (9)	0.50255 (7)	0.0223 (2)	
N4A	0.59586 (9)	0.60225 (8)	0.42386 (7)	0.0212 (2)	
N5A	0.75776 (9)	0.56285 (9)	0.34326 (8)	0.0242 (2)	
H4AB	0.6995	0.5513	0.2983	0.029*	
C1A	0.50547 (10)	0.58304 (9)	0.35331 (8)	0.0196 (2)	
C2A	0.43486 (11)	0.64838 (9)	0.47960 (8)	0.0201 (2)	
C3A	0.34541 (11)	0.69354 (10)	0.53838 (9)	0.0237 (3)	
H4AA	0.2738	0.6391	0.5371	0.028*	
C4A	0.30062 (13)	0.80053 (12)	0.50000 (10)	0.0315 (3)	
H5AA	0.2599	0.7854	0.4387	0.047*	
H5AB	0.3704	0.8535	0.4979	0.047*	
H5AC	0.2429	0.8309	0.5389	0.047*	
C5A	0.40205 (14)	0.71189 (12)	0.63627 (9)	0.0305 (3)	
H6AA	0.4295	0.6423	0.6590	0.046*	
H6AB	0.3411	0.7393	0.6737	0.046*	
H6AC	0.4719	0.7658	0.6389	0.046*	
C6A	0.72576 (11)	0.59224 (10)	0.42504 (9)	0.0233 (2)	
C7A	0.88576 (11)	0.54861 (11)	0.32375 (10)	0.0271 (3)	
C8A	0.96498 (13)	0.65373 (13)	0.34987 (12)	0.0372 (3)	
H9AA	0.9690	0.6685	0.4155	0.056*	
H9AB	0.9294	0.7151	0.3184	0.056*	
H9AC	1.0474	0.6451	0.3325	0.056*	
C9A	0.93520 (13)	0.45147 (13)	0.37431 (11)	0.0363 (3)	
H10A	0.9283	0.4631	0.4392	0.054*	
H10B	1.0211	0.4452	0.3644	0.054*	
H10C	0.8879	0.3838	0.3517	0.054*	
C10A	0.87979 (14)	0.52432 (15)	0.22191 (11)	0.0389 (3)	
H11A	0.8453	0.5857	0.1898	0.058*	
H11B	0.8281	0.4571	0.2055	0.058*	
H11C	0.9622	0.5145	0.2049	0.058*	
O1B	0.32055 (8)	0.95985 (7)	0.03184 (6)	0.02541 (19)	
O2B	0.15234 (8)	0.69360 (7)	0.16012 (6)	0.0269 (2)	
N1B	0.46600 (9)	0.93184 (8)	0.15285 (7)	0.0201 (2)	
N2B	0.54880 (10)	1.02214 (9)	0.14557 (8)	0.0242 (2)	
H3B2	0.5068 (16)	1.0854 (15)	0.1505 (12)	0.030 (4)*	
H3B1	0.5749 (17)	1.0154 (15)	0.0910 (13)	0.034 (4)*	
N3B	0.37300 (9)	0.79451 (8)	0.21715 (7)	0.0221 (2)	
N4B	0.30260 (9)	0.82468 (8)	0.13967 (7)	0.0211 (2)	
N5B	0.12373 (10)	0.81232 (9)	0.04498 (7)	0.0258 (2)	
H4BB	0.1620	0.8634	0.0162	0.031*	
C1B	0.35739 (11)	0.91208 (10)	0.09966 (8)	0.0206 (2)	
C2B	0.46927 (11)	0.86178 (10)	0.22317 (8)	0.0208 (2)	
C3B	0.56869 (12)	0.86298 (10)	0.29937 (9)	0.0261 (3)	
H4BA	0.5476	0.8016	0.3388	0.031*	
C4B	0.69240 (13)	0.84177 (13)	0.26659 (12)	0.0381 (3)	
H5BA	0.6867	0.7711	0.2323	0.057*	
H5BB	0.7538	0.8401	0.3189	0.057*	
H5BC	0.7161	0.9005	0.2275	0.057*	
C5B	0.57330 (15)	0.96978 (12)	0.35741 (10)	0.0354 (3)	
H6BA	0.4919	0.9814	0.3757	0.053*	
H6BB	0.5999	1.0314	0.3222	0.053*	
H6BC	0.6311	0.9649	0.4114	0.053*	
C6B	0.18508 (11)	0.77003 (10)	0.11635 (8)	0.0214 (2)	
C7B	−0.00412 (14)	0.77871 (12)	0.01138 (11)	0.0363 (3)	
C8B	−0.08729 (15)	0.80880 (16)	0.08385 (15)	0.0514 (5)	
H9BA	−0.0789	0.8883	0.0972	0.077*	
H9BB	−0.0640	0.7705	0.1391	0.077*	
H9BC	−0.1723	0.7869	0.0617	0.077*	
C9B	−0.0174 (2)	0.65528 (15)	−0.01308 (14)	0.0556 (5)	
H10D	0.0374	0.6378	−0.0589	0.083*	
H10E	−0.1019	0.6345	−0.0372	0.083*	
H10F	0.0038	0.6145	0.0412	0.083*	
C10B	−0.0332 (2)	0.84417 (17)	−0.07255 (14)	0.0600 (6)	
H11D	0.0175	0.8216	−0.1197	0.090*	
H11E	−0.0162	0.9227	−0.0570	0.090*	
H11F	−0.1196	0.8303	−0.0948	0.090*	
O1C	0.18247 (8)	0.38918 (7)	0.24910 (6)	0.02389 (19)	
O2C	0.32253 (9)	0.07163 (7)	0.25786 (7)	0.0297 (2)	
N1C	0.33153 (9)	0.40112 (8)	0.14853 (7)	0.0196 (2)	
N2C	0.32178 (11)	0.51004 (9)	0.12243 (8)	0.0232 (2)	
H3C2	0.3638 (16)	0.5511 (15)	0.1687 (12)	0.030 (4)*	
H3C1	0.2454 (19)	0.5268 (16)	0.1224 (13)	0.039 (5)*	
N3C	0.40591 (9)	0.23804 (9)	0.16020 (7)	0.0222 (2)	
N4C	0.31110 (9)	0.24896 (8)	0.21498 (7)	0.0200 (2)	
N5C	0.17403 (10)	0.17616 (8)	0.30715 (7)	0.0227 (2)	
H4CB	0.1416	0.2398	0.3002	0.027*	
C1C	0.26465 (11)	0.35096 (10)	0.21013 (8)	0.0192 (2)	
C2C	0.41530 (11)	0.33083 (10)	0.12170 (8)	0.0201 (2)	
C3C	0.50113 (12)	0.35820 (10)	0.05267 (8)	0.0234 (2)	
H4CA	0.5188	0.4396	0.0556	0.028*	
C4C	0.62141 (13)	0.30430 (13)	0.07196 (11)	0.0349 (3)	
H5CA	0.6552	0.3228	0.1346	0.052*	
H5CB	0.6792	0.3309	0.0305	0.052*	
H5CC	0.6073	0.2245	0.0632	0.052*	
C5C	0.43789 (14)	0.32422 (13)	−0.04145 (9)	0.0346 (3)	
H6CA	0.3638	0.3642	−0.0527	0.052*	
H6CB	0.4160	0.2451	−0.0447	0.052*	
H6CC	0.4933	0.3415	−0.0873	0.052*	
C6C	0.27048 (11)	0.15583 (10)	0.26283 (8)	0.0208 (2)	
C7C	0.11856 (12)	0.09791 (10)	0.36725 (9)	0.0257 (3)	
C8C	0.07780 (17)	−0.01067 (13)	0.31583 (12)	0.0425 (4)	
H9CA	0.1492	−0.0449	0.2957	0.064*	
H9CB	0.0209	0.0029	0.2631	0.064*	
H9CC	0.0371	−0.0597	0.3557	0.064*	
C9C	0.21050 (15)	0.08115 (13)	0.44839 (10)	0.0356 (3)	
H10G	0.2817	0.0472	0.4276	0.053*	
H10H	0.1725	0.0331	0.4904	0.053*	
H10I	0.2363	0.1523	0.4793	0.053*	
C10C	0.00908 (15)	0.15331 (13)	0.39930 (12)	0.0402 (4)	
H11G	0.0366	0.2242	0.4299	0.060*	
H11H	−0.0301	0.1064	0.4415	0.060*	
H11I	−0.0496	0.1649	0.3470	0.060*	

### Atomic displacement parameters (Å^2^)

**Table:**

	*U*^11^	*U*^22^	*U*^33^	*U*^12^	*U*^13^	*U*^23^
O1A	0.0203 (4)	0.0274 (4)	0.0250 (4)	0.0057 (3)	0.0033 (3)	−0.0002 (3)
O2A	0.0208 (4)	0.0459 (6)	0.0339 (5)	0.0082 (4)	−0.0026 (4)	−0.0038 (4)
N1A	0.0158 (5)	0.0186 (5)	0.0244 (5)	0.0027 (4)	0.0026 (4)	0.0020 (4)
N2A	0.0144 (5)	0.0250 (5)	0.0287 (5)	0.0030 (4)	0.0003 (4)	0.0006 (4)
N3A	0.0208 (5)	0.0224 (5)	0.0246 (5)	0.0050 (4)	0.0040 (4)	0.0009 (4)
N4A	0.0171 (5)	0.0221 (5)	0.0249 (5)	0.0052 (4)	0.0035 (4)	0.0010 (4)
N5A	0.0155 (5)	0.0280 (5)	0.0297 (5)	0.0044 (4)	0.0032 (4)	0.0028 (4)
C1A	0.0166 (5)	0.0164 (5)	0.0266 (6)	0.0031 (4)	0.0036 (4)	0.0045 (4)
C2A	0.0208 (6)	0.0157 (5)	0.0247 (6)	0.0026 (4)	0.0038 (4)	0.0037 (4)
C3A	0.0217 (6)	0.0226 (6)	0.0280 (6)	0.0032 (5)	0.0073 (5)	0.0004 (5)
C4A	0.0333 (7)	0.0283 (7)	0.0341 (7)	0.0140 (5)	0.0036 (6)	−0.0015 (5)
C5A	0.0368 (7)	0.0288 (7)	0.0272 (6)	0.0071 (5)	0.0069 (5)	0.0009 (5)
C6A	0.0160 (6)	0.0211 (6)	0.0332 (7)	0.0050 (4)	0.0022 (5)	0.0036 (5)
C7A	0.0173 (6)	0.0293 (7)	0.0365 (7)	0.0057 (5)	0.0075 (5)	0.0044 (5)
C8A	0.0201 (6)	0.0352 (8)	0.0571 (9)	0.0007 (5)	0.0088 (6)	0.0016 (7)
C9A	0.0255 (7)	0.0365 (8)	0.0501 (9)	0.0132 (6)	0.0109 (6)	0.0102 (6)
C10A	0.0283 (7)	0.0515 (9)	0.0388 (8)	0.0049 (6)	0.0124 (6)	0.0011 (7)
O1B	0.0264 (4)	0.0276 (5)	0.0227 (4)	0.0022 (4)	0.0015 (3)	0.0088 (3)
O2B	0.0252 (4)	0.0241 (4)	0.0314 (5)	−0.0006 (3)	0.0015 (4)	0.0074 (4)
N1B	0.0200 (5)	0.0179 (5)	0.0230 (5)	0.0025 (4)	0.0027 (4)	0.0027 (4)
N2B	0.0236 (5)	0.0193 (5)	0.0302 (6)	−0.0007 (4)	0.0045 (4)	0.0040 (4)
N3B	0.0222 (5)	0.0221 (5)	0.0220 (5)	0.0042 (4)	−0.0005 (4)	0.0051 (4)
N4B	0.0208 (5)	0.0220 (5)	0.0205 (5)	0.0020 (4)	0.0003 (4)	0.0052 (4)
N5B	0.0237 (5)	0.0282 (5)	0.0249 (5)	−0.0014 (4)	−0.0011 (4)	0.0062 (4)
C1B	0.0211 (6)	0.0202 (5)	0.0214 (5)	0.0038 (4)	0.0051 (4)	0.0017 (4)
C2B	0.0222 (6)	0.0177 (5)	0.0231 (6)	0.0054 (4)	0.0024 (4)	0.0024 (4)
C3B	0.0265 (6)	0.0220 (6)	0.0286 (6)	0.0036 (5)	−0.0052 (5)	0.0040 (5)
C4B	0.0259 (7)	0.0384 (8)	0.0491 (9)	0.0114 (6)	−0.0060 (6)	0.0020 (6)
C5B	0.0417 (8)	0.0307 (7)	0.0311 (7)	0.0070 (6)	−0.0101 (6)	−0.0026 (6)
C6B	0.0212 (6)	0.0212 (6)	0.0219 (6)	0.0026 (4)	0.0029 (4)	0.0006 (4)
C7B	0.0317 (7)	0.0334 (7)	0.0396 (8)	−0.0063 (6)	−0.0143 (6)	0.0074 (6)
C8B	0.0243 (7)	0.0490 (10)	0.0815 (14)	0.0017 (7)	0.0040 (8)	0.0148 (9)
C9B	0.0708 (13)	0.0385 (9)	0.0495 (10)	−0.0147 (8)	−0.0227 (9)	−0.0014 (7)
C10B	0.0620 (12)	0.0555 (11)	0.0538 (11)	−0.0152 (9)	−0.0334 (9)	0.0199 (9)
O1C	0.0243 (4)	0.0211 (4)	0.0284 (4)	0.0056 (3)	0.0095 (3)	0.0046 (3)
O2C	0.0361 (5)	0.0217 (4)	0.0348 (5)	0.0100 (4)	0.0133 (4)	0.0084 (4)
N1C	0.0209 (5)	0.0180 (5)	0.0207 (5)	0.0024 (4)	0.0038 (4)	0.0036 (4)
N2C	0.0261 (6)	0.0181 (5)	0.0265 (5)	0.0040 (4)	0.0047 (4)	0.0066 (4)
N3C	0.0222 (5)	0.0221 (5)	0.0240 (5)	0.0036 (4)	0.0085 (4)	0.0023 (4)
N4C	0.0205 (5)	0.0195 (5)	0.0213 (5)	0.0038 (4)	0.0060 (4)	0.0036 (4)
N5C	0.0254 (5)	0.0197 (5)	0.0252 (5)	0.0052 (4)	0.0074 (4)	0.0081 (4)
C1C	0.0199 (5)	0.0187 (5)	0.0187 (5)	0.0009 (4)	0.0006 (4)	0.0027 (4)
C2C	0.0204 (6)	0.0198 (5)	0.0202 (5)	0.0017 (4)	0.0022 (4)	0.0003 (4)
C3C	0.0261 (6)	0.0207 (6)	0.0245 (6)	−0.0005 (5)	0.0091 (5)	0.0019 (4)
C4C	0.0281 (7)	0.0365 (7)	0.0432 (8)	0.0047 (6)	0.0152 (6)	0.0083 (6)
C5C	0.0383 (8)	0.0413 (8)	0.0243 (6)	−0.0086 (6)	0.0101 (6)	0.0004 (6)
C6C	0.0244 (6)	0.0193 (6)	0.0189 (5)	0.0021 (4)	0.0016 (4)	0.0045 (4)
C7C	0.0281 (6)	0.0231 (6)	0.0281 (6)	0.0027 (5)	0.0094 (5)	0.0098 (5)
C8C	0.0496 (9)	0.0309 (8)	0.0464 (9)	−0.0112 (7)	0.0099 (7)	0.0037 (6)
C9C	0.0412 (8)	0.0385 (8)	0.0292 (7)	0.0065 (6)	0.0063 (6)	0.0150 (6)
C10C	0.0350 (8)	0.0403 (8)	0.0512 (9)	0.0100 (6)	0.0222 (7)	0.0198 (7)

### Geometric parameters (Å, º)

**Table:**

O1A—C1A	1.2329 (15)	C4B—H5BA	0.9800
O2A—C6A	1.2061 (16)	C4B—H5BB	0.9800
N1A—C1A	1.3725 (15)	C4B—H5BC	0.9800
N1A—C2A	1.3764 (16)	C5B—H6BA	0.9800
N1A—N2A	1.4028 (14)	C5B—H6BB	0.9800
N2A—H3N1	0.904 (19)	C5B—H6BC	0.9800
N2A—H3N2	0.91 (2)	C7B—C10B	1.525 (2)
N3A—C2A	1.2971 (16)	C7B—C8B	1.530 (3)
N3A—N4A	1.4004 (14)	C7B—C9B	1.532 (2)
N4A—C1A	1.3757 (16)	C8B—H9BA	0.9800
N4A—C6A	1.4435 (15)	C8B—H9BB	0.9800
N5A—C6A	1.3398 (17)	C8B—H9BC	0.9800
N5A—C7A	1.4879 (15)	C9B—H10D	0.9800
N5A—H4AB	0.8800	C9B—H10E	0.9800
C2A—C3A	1.4959 (16)	C9B—H10F	0.9800
C3A—C5A	1.5271 (19)	C10B—H11D	0.9800
C3A—C4A	1.5346 (18)	C10B—H11E	0.9800
C3A—H4AA	1.0000	C10B—H11F	0.9800
C4A—H5AA	0.9800	O1C—C1C	1.2278 (15)
C4A—H5AB	0.9800	O2C—C6C	1.2103 (16)
C4A—H5AC	0.9800	N1C—C1C	1.3717 (15)
C5A—H6AA	0.9800	N1C—C2C	1.3746 (16)
C5A—H6AB	0.9800	N1C—N2C	1.4072 (14)
C5A—H6AC	0.9800	N2C—H3C2	0.906 (18)
C7A—C10A	1.524 (2)	N2C—H3C1	0.88 (2)
C7A—C8A	1.524 (2)	N3C—C2C	1.2926 (16)
C7A—C9A	1.5276 (19)	N3C—N4C	1.3967 (14)
C8A—H9AA	0.9800	N4C—C1C	1.3764 (15)
C8A—H9AB	0.9800	N4C—C6C	1.4373 (15)
C8A—H9AC	0.9800	N5C—C6C	1.3358 (16)
C9A—H10A	0.9800	N5C—C7C	1.4802 (15)
C9A—H10B	0.9800	N5C—H4CB	0.8800
C9A—H10C	0.9800	C2C—C3C	1.4972 (16)
C10A—H11A	0.9800	C3C—C4C	1.5224 (19)
C10A—H11B	0.9800	C3C—C5C	1.5298 (19)
C10A—H11C	0.9800	C3C—H4CA	1.0000
O1B—C1B	1.2301 (15)	C4C—H5CA	0.9800
O2B—C6B	1.2161 (15)	C4C—H5CB	0.9800
N1B—C1B	1.3713 (16)	C4C—H5CC	0.9800
N1B—C2B	1.3750 (15)	C5C—H6CA	0.9800
N1B—N2B	1.4044 (14)	C5C—H6CB	0.9800
N2B—H3B2	0.929 (19)	C5C—H6CC	0.9800
N2B—H3B1	0.89 (2)	C7C—C9C	1.5243 (19)
N3B—C2B	1.2973 (17)	C7C—C8C	1.525 (2)
N3B—N4B	1.3974 (14)	C7C—C10C	1.5258 (19)
N4B—C1B	1.3794 (16)	C8C—H9CA	0.9800
N4B—C6B	1.4270 (16)	C8C—H9CB	0.9800
N5B—C6B	1.3380 (16)	C8C—H9CC	0.9800
N5B—C7B	1.4771 (17)	C9C—H10G	0.9800
N5B—H4BB	0.8800	C9C—H10H	0.9800
C2B—C3B	1.4948 (17)	C9C—H10I	0.9800
C3B—C4B	1.526 (2)	C10C—H11G	0.9800
C3B—C5B	1.5306 (19)	C10C—H11H	0.9800
C3B—H4BA	1.0000	C10C—H11I	0.9800
			
C1A—N1A—C2A	109.01 (10)	C3B—C5B—H6BB	109.5
C1A—N1A—N2A	126.31 (10)	H6BA—C5B—H6BB	109.5
C2A—N1A—N2A	124.69 (10)	C3B—C5B—H6BC	109.5
N1A—N2A—H3N1	107.3 (11)	H6BA—C5B—H6BC	109.5
N1A—N2A—H3N2	109.0 (12)	H6BB—C5B—H6BC	109.5
H3N1—N2A—H3N2	114.1 (16)	O2B—C6B—N5B	127.90 (12)
C2A—N3A—N4A	104.12 (10)	O2B—C6B—N4B	119.55 (11)
C1A—N4A—N3A	112.25 (9)	N5B—C6B—N4B	112.54 (10)
C1A—N4A—C6A	129.36 (10)	N5B—C7B—C10B	105.72 (13)
N3A—N4A—C6A	118.27 (10)	N5B—C7B—C8B	109.22 (13)
C6A—N5A—C7A	123.99 (11)	C10B—C7B—C8B	110.02 (16)
C6A—N5A—H4AB	118.0	N5B—C7B—C9B	110.28 (14)
C7A—N5A—H4AB	118.0	C10B—C7B—C9B	110.52 (15)
O1A—C1A—N1A	127.90 (11)	C8B—C7B—C9B	110.95 (15)
O1A—C1A—N4A	129.20 (11)	C7B—C8B—H9BA	109.5
N1A—C1A—N4A	102.90 (10)	C7B—C8B—H9BB	109.5
N3A—C2A—N1A	111.70 (10)	H9BA—C8B—H9BB	109.5
N3A—C2A—C3A	125.55 (11)	C7B—C8B—H9BC	109.5
N1A—C2A—C3A	122.66 (11)	H9BA—C8B—H9BC	109.5
C2A—C3A—C5A	111.00 (11)	H9BB—C8B—H9BC	109.5
C2A—C3A—C4A	109.25 (10)	C7B—C9B—H10D	109.5
C5A—C3A—C4A	111.24 (11)	C7B—C9B—H10E	109.5
C2A—C3A—H4AA	108.4	H10D—C9B—H10E	109.5
C5A—C3A—H4AA	108.4	C7B—C9B—H10F	109.5
C4A—C3A—H4AA	108.4	H10D—C9B—H10F	109.5
C3A—C4A—H5AA	109.5	H10E—C9B—H10F	109.5
C3A—C4A—H5AB	109.5	C7B—C10B—H11D	109.5
H5AA—C4A—H5AB	109.5	C7B—C10B—H11E	109.5
C3A—C4A—H5AC	109.5	H11D—C10B—H11E	109.5
H5AA—C4A—H5AC	109.5	C7B—C10B—H11F	109.5
H5AB—C4A—H5AC	109.5	H11D—C10B—H11F	109.5
C3A—C5A—H6AA	109.5	H11E—C10B—H11F	109.5
C3A—C5A—H6AB	109.5	C1C—N1C—C2C	109.08 (10)
H6AA—C5A—H6AB	109.5	C1C—N1C—N2C	125.18 (10)
C3A—C5A—H6AC	109.5	C2C—N1C—N2C	125.58 (10)
H6AA—C5A—H6AC	109.5	N1C—N2C—H3C2	104.3 (11)
H6AB—C5A—H6AC	109.5	N1C—N2C—H3C1	108.9 (12)
O2A—C6A—N5A	128.31 (12)	H3C2—N2C—H3C1	105.8 (17)
O2A—C6A—N4A	119.58 (12)	C2C—N3C—N4C	104.58 (10)
N5A—C6A—N4A	112.11 (11)	C1C—N4C—N3C	111.95 (9)
N5A—C7A—C10A	105.80 (11)	C1C—N4C—C6C	128.99 (10)
N5A—C7A—C8A	110.33 (11)	N3C—N4C—C6C	118.94 (10)
C10A—C7A—C8A	109.89 (12)	C6C—N5C—C7C	123.83 (10)
N5A—C7A—C9A	109.66 (11)	C6C—N5C—H4CB	118.1
C10A—C7A—C9A	109.83 (12)	C7C—N5C—H4CB	118.1
C8A—C7A—C9A	111.18 (12)	O1C—C1C—N1C	127.35 (11)
C7A—C8A—H9AA	109.5	O1C—C1C—N4C	129.76 (11)
C7A—C8A—H9AB	109.5	N1C—C1C—N4C	102.87 (10)
H9AA—C8A—H9AB	109.5	N3C—C2C—N1C	111.48 (10)
C7A—C8A—H9AC	109.5	N3C—C2C—C3C	125.19 (11)
H9AA—C8A—H9AC	109.5	N1C—C2C—C3C	123.25 (11)
H9AB—C8A—H9AC	109.5	C2C—C3C—C4C	111.38 (11)
C7A—C9A—H10A	109.5	C2C—C3C—C5C	108.67 (10)
C7A—C9A—H10B	109.5	C4C—C3C—C5C	112.05 (12)
H10A—C9A—H10B	109.5	C2C—C3C—H4CA	108.2
C7A—C9A—H10C	109.5	C4C—C3C—H4CA	108.2
H10A—C9A—H10C	109.5	C5C—C3C—H4CA	108.2
H10B—C9A—H10C	109.5	C3C—C4C—H5CA	109.5
C7A—C10A—H11A	109.5	C3C—C4C—H5CB	109.5
C7A—C10A—H11B	109.5	H5CA—C4C—H5CB	109.5
H11A—C10A—H11B	109.5	C3C—C4C—H5CC	109.5
C7A—C10A—H11C	109.5	H5CA—C4C—H5CC	109.5
H11A—C10A—H11C	109.5	H5CB—C4C—H5CC	109.5
H11B—C10A—H11C	109.5	C3C—C5C—H6CA	109.5
C1B—N1B—C2B	108.91 (10)	C3C—C5C—H6CB	109.5
C1B—N1B—N2B	124.54 (10)	H6CA—C5C—H6CB	109.5
C2B—N1B—N2B	125.57 (10)	C3C—C5C—H6CC	109.5
N1B—N2B—H3B2	107.8 (11)	H6CA—C5C—H6CC	109.5
N1B—N2B—H3B1	106.6 (12)	H6CB—C5C—H6CC	109.5
H3B2—N2B—H3B1	110.3 (16)	O2C—C6C—N5C	128.46 (11)
C2B—N3B—N4B	104.24 (10)	O2C—C6C—N4C	119.36 (11)
C1B—N4B—N3B	112.05 (10)	N5C—C6C—N4C	112.16 (10)
C1B—N4B—C6B	129.10 (10)	N5C—C7C—C9C	109.05 (11)
N3B—N4B—C6B	118.67 (10)	N5C—C7C—C8C	110.71 (11)
C6B—N5B—C7B	124.13 (11)	C9C—C7C—C8C	111.28 (12)
C6B—N5B—H4BB	117.9	N5C—C7C—C10C	105.50 (10)
C7B—N5B—H4BB	117.9	C9C—C7C—C10C	109.49 (12)
O1B—C1B—N1B	127.81 (11)	C8C—C7C—C10C	110.63 (13)
O1B—C1B—N4B	129.22 (11)	C7C—C8C—H9CA	109.5
N1B—C1B—N4B	102.95 (10)	C7C—C8C—H9CB	109.5
N3B—C2B—N1B	111.74 (11)	H9CA—C8C—H9CB	109.5
N3B—C2B—C3B	123.07 (11)	C7C—C8C—H9CC	109.5
N1B—C2B—C3B	125.18 (11)	H9CA—C8C—H9CC	109.5
C2B—C3B—C4B	112.37 (11)	H9CB—C8C—H9CC	109.5
C2B—C3B—C5B	110.69 (11)	C7C—C9C—H10G	109.5
C4B—C3B—C5B	111.95 (12)	C7C—C9C—H10H	109.5
C2B—C3B—H4BA	107.2	H10G—C9C—H10H	109.5
C4B—C3B—H4BA	107.2	C7C—C9C—H10I	109.5
C5B—C3B—H4BA	107.2	H10G—C9C—H10I	109.5
C3B—C4B—H5BA	109.5	H10H—C9C—H10I	109.5
C3B—C4B—H5BB	109.5	C7C—C10C—H11G	109.5
H5BA—C4B—H5BB	109.5	C7C—C10C—H11H	109.5
C3B—C4B—H5BC	109.5	H11G—C10C—H11H	109.5
H5BA—C4B—H5BC	109.5	C7C—C10C—H11I	109.5
H5BB—C4B—H5BC	109.5	H11G—C10C—H11I	109.5
C3B—C5B—H6BA	109.5	H11H—C10C—H11I	109.5
			
C2A—N3A—N4A—C1A	0.10 (13)	N2B—N1B—C2B—C3B	−6.47 (19)
C2A—N3A—N4A—C6A	176.55 (10)	N3B—C2B—C3B—C4B	120.86 (14)
C2A—N1A—C1A—O1A	−178.86 (12)	N1B—C2B—C3B—C4B	−60.80 (16)
N2A—N1A—C1A—O1A	0.90 (19)	N3B—C2B—C3B—C5B	−113.15 (14)
C2A—N1A—C1A—N4A	0.84 (12)	N1B—C2B—C3B—C5B	65.18 (17)
N2A—N1A—C1A—N4A	−179.39 (10)	C7B—N5B—C6B—O2B	6.4 (2)
N3A—N4A—C1A—O1A	179.11 (11)	C7B—N5B—C6B—N4B	−174.12 (12)
C6A—N4A—C1A—O1A	3.2 (2)	C1B—N4B—C6B—O2B	179.99 (12)
N3A—N4A—C1A—N1A	−0.59 (12)	N3B—N4B—C6B—O2B	−5.26 (17)
C6A—N4A—C1A—N1A	−176.55 (11)	C1B—N4B—C6B—N5B	0.45 (18)
N4A—N3A—C2A—N1A	0.46 (13)	N3B—N4B—C6B—N5B	175.21 (10)
N4A—N3A—C2A—C3A	−176.14 (11)	C6B—N5B—C7B—C10B	−177.48 (15)
C1A—N1A—C2A—N3A	−0.86 (13)	C6B—N5B—C7B—C8B	64.18 (17)
N2A—N1A—C2A—N3A	179.37 (10)	C6B—N5B—C7B—C9B	−57.99 (19)
C1A—N1A—C2A—C3A	175.85 (10)	C2C—N3C—N4C—C1C	−1.08 (13)
N2A—N1A—C2A—C3A	−3.92 (17)	C2C—N3C—N4C—C6C	175.35 (10)
N3A—C2A—C3A—C5A	−11.65 (17)	C2C—N1C—C1C—O1C	179.41 (12)
N1A—C2A—C3A—C5A	172.11 (11)	N2C—N1C—C1C—O1C	3.8 (2)
N3A—C2A—C3A—C4A	111.38 (14)	C2C—N1C—C1C—N4C	−1.73 (12)
N1A—C2A—C3A—C4A	−64.86 (15)	N2C—N1C—C1C—N4C	−177.36 (10)
C7A—N5A—C6A—O2A	−1.1 (2)	N3C—N4C—C1C—O1C	−179.44 (12)
C7A—N5A—C6A—N4A	178.68 (11)	C6C—N4C—C1C—O1C	4.6 (2)
C1A—N4A—C6A—O2A	−178.10 (12)	N3C—N4C—C1C—N1C	1.74 (13)
N3A—N4A—C6A—O2A	6.15 (17)	C6C—N4C—C1C—N1C	−174.23 (11)
C1A—N4A—C6A—N5A	2.10 (18)	N4C—N3C—C2C—N1C	−0.09 (13)
N3A—N4A—C6A—N5A	−173.65 (10)	N4C—N3C—C2C—C3C	−177.01 (11)
C6A—N5A—C7A—C10A	−175.58 (12)	C1C—N1C—C2C—N3C	1.21 (14)
C6A—N5A—C7A—C8A	−56.76 (17)	N2C—N1C—C2C—N3C	176.81 (11)
C6A—N5A—C7A—C9A	66.03 (16)	C1C—N1C—C2C—C3C	178.20 (11)
C2B—N3B—N4B—C1B	−0.70 (13)	N2C—N1C—C2C—C3C	−6.20 (18)
C2B—N3B—N4B—C6B	−176.31 (10)	N3C—C2C—C3C—C4C	−35.08 (17)
C2B—N1B—C1B—O1B	178.39 (12)	N1C—C2C—C3C—C4C	148.34 (12)
N2B—N1B—C1B—O1B	9.2 (2)	N3C—C2C—C3C—C5C	88.83 (15)
C2B—N1B—C1B—N4B	−3.08 (12)	N1C—C2C—C3C—C5C	−87.75 (14)
N2B—N1B—C1B—N4B	−172.31 (10)	C7C—N5C—C6C—O2C	5.9 (2)
N3B—N4B—C1B—O1B	−179.14 (12)	C7C—N5C—C6C—N4C	−175.50 (11)
C6B—N4B—C1B—O1B	−4.1 (2)	C1C—N4C—C6C—O2C	178.93 (12)
N3B—N4B—C1B—N1B	2.37 (13)	N3C—N4C—C6C—O2C	3.20 (17)
C6B—N4B—C1B—N1B	177.41 (11)	C1C—N4C—C6C—N5C	0.15 (18)
N4B—N3B—C2B—N1B	−1.35 (13)	N3C—N4C—C6C—N5C	−175.58 (10)
N4B—N3B—C2B—C3B	177.19 (11)	C6C—N5C—C7C—C9C	64.87 (16)
C1B—N1B—C2B—N3B	2.95 (14)	C6C—N5C—C7C—C8C	−57.90 (17)
N2B—N1B—C2B—N3B	172.03 (11)	C6C—N5C—C7C—C10C	−177.61 (13)
C1B—N1B—C2B—C3B	−175.55 (11)		

### Hydrogen-bond geometry (Å, º)

**Table:**

*D*—H···*A*	*D*—H	H···*A*	*D*···*A*	*D*—H···*A*
N2*A*—H3*N*1···O1*C*	0.904 (19)	2.220 (19)	3.0832 (14)	159.6 (15)
N2*A*—H3*N*2···N3*B*	0.91 (2)	2.40 (2)	3.2251 (15)	150.9 (15)
N2*A*—H3*N*2···O2*B*	0.91 (2)	2.483 (19)	3.2492 (14)	141.7 (15)
N5*A*—H4*AB*···O1*A*	0.88	2.05	2.7770 (14)	140
N2*B*—H3*B*2···N3*C*^i^	0.929 (19)	2.233 (19)	3.1619 (15)	179.1 (15)
N2*B*—H3*B*1···O1*B*^ii^	0.89 (2)	2.27 (2)	3.1356 (15)	163.1 (16)
N5*B*—H4*BB*···O1*B*	0.88	2.04	2.7655 (14)	139
N2*C*—H3*C*2···O1*A*	0.906 (18)	2.171 (18)	2.9455 (15)	143.1 (15)
N2*C*—H3*C*1···O2*B*	0.88 (2)	2.41 (2)	3.0718 (14)	132.8 (16)
N5*C*—H4*CB*···O1*C*	0.88	2.05	2.7721 (13)	139

Symmetry codes: (i) *x*, *y*+1, *z*; (ii) −*x*+1, −*y*+2, −*z*.
